# Experimental Investigation and Comparative Analysis of Aluminium Hybrid Metal Matrix Composites Reinforced with Silicon Nitride, Eggshell and Magnesium

**DOI:** 10.3390/ma15176098

**Published:** 2022-09-02

**Authors:** Dhanenthiran Mohan, Balamurugan Chinnasamy, Senthil Kumar Naganathan, Nagaprasad Nagaraj, LetaTesfaye Jule, Bayissa Badassa, Krishnaraj Ramaswamy, Parthiban Kathirvel, Gunasekaran Murali, Nikolai Ivanovich Vatin

**Affiliations:** 1 Department of Mechanical Engineering, SRM TRP Engineering College, Trichy 621105, India; 2 Department of Mechanical Engineering, College of Engineering Guindy, Anna University, Chennai 600025, India; 3 Department of Mechanical Engineering, ULTRA College of Engineering and Technology, Madurai 625104, India; 4 Centre for Excellence-Indigenous Knowledge, Innovative Technology Transfer and Entrepreneurship, Dambi Dollo University, Dembi Dolo 260, Ethiopia; 5 Department of Physics, College of Natural and Computational Science, Dambi Dollo University, Dembi Dolo 260, Ethiopia; 6 Ministry of Innovation and Technology, Addis Ababa 260, Ethiopia; 7 Department of Mechanical Engineering, Dambi Dollo University, Dembi Dolo 260, Ethiopia; 8 School of Civil Engineering, SASTRA Deemed University, Thanjavur 613401, India; 9 Peter the Great St. Petersburg Polytechnic University, Saint Petersburg 195251, Russia; 10 Division of Research and Innovation, Uttaranchal University, Dehradun 248007, India

**Keywords:** metal matrix composites (MMCs), reinforcement, mechanical properties, machining parameters, optimisation techniques, ANSYS prediction

## Abstract

In today’s scenario, composite materials play a vital role in automobile, aerospace, and defence sectors because of their higher strength, light weight and other mechanical properties. Aluminium alloy Al6082 is a medium strength alloy with good corrosion resistance properties; hence, it is used for high-stress applications, bridges, cranes, etc. The present work focuses on comparing the mechanical properties of Al6082 and Al6082 with the addition of silicon nitride, magnesium, and bio waste of eggshells. Samples of Al6082 reinforced with 2% of silicon nitride (Si_3_N_4_), 5% of eggshell, and 1% magnesium reinforcements were prepared using the crucible casting process. Mechanical properties were evaluated through hardness test, tensile test and compressive tests, which varied with the additives of reinforcement materials. The results showed that the reinforced materials could increase mechanical properties. Further, it will be analysed by the machining parameters involved through the CNC turning process. Analysis of variance from optimisation technique shows a noteworthy increment of material removal rate (MRR) and significant decrement in surface roughness (Ra) and machining time compared to standard aluminium. Additionally, the analysis of mechanical testing has been predicted with the outcomes of stresses and displacements using the ANSYS platform.

## 1. Introduction

Composites are just a variety of materials mixed so that the resulting materials have the required properties. Nowadays, composite material usage includes more applications such as aeronautics, marine application, sports, etc. Metal matrix composites, especially aluminium-based metal matrix composites, obtain the most applications in the present day. The addition of silicon nitride and eggshell waste created from domestic’s usage improves the mechanical properties of the materials [1,2,3,4,5]. Shashi Prakash Dwivedi et al. (2019) found that composite material preparation using AA2014 with uncarbonised eggshell powder particles by electromagnetic stir casting techniques gives high hardness. Gururaj Parande et al. (2019) analysed the Mg-Zn-based aluminium composites to enhance the superior grain refining ability, damping, and mechanical properties. Soutrik Bose et al. (2018) found that waste carbonised eggshell reinforced aluminium alloy gave better tribo-mechanical properties and decreased the corrosion resistance rate. Vikas Verma et al. (2018) concluded that the addition of eggshell particles in Al6061 gives better results in mechanical properties. Shahrukh Shamim et al. (2016) found that eggshells successfully incorporated with Al–Si-Mg-Ti alloys and dispersed the CaO, which increased the yield strength and tensile strength [6,7,8,9,10]. Johnson O. Agunsoye et al. (2016) justified that 100 micron meter CaCO_3_ particles are successfully incorporated with Aluminium material, giving high tensile strain. A.R. Sivaram et al. (2015) found that reinforcement of aluminium alloy mixed with zirconium oxide increases creep’s strength and load-displacement. Viney Kumar et al. (2014) found that Al6061 reinforced with different percentages of fly ash gives a better tensile strength compared to an addition of Al6061 reinforced with graphite. At the same time, the author found that the addition of graphite decreases the mechanical properties. Amba Chaithanyasai et al. (2014) found that eggshell particles distribute evenly in aluminium alloy, and hardness improvement of about 14% compared to aluminium alloy. S.B. Chikalthankar et al. (2014) found that 12% of Sic composition has higher resistance, and SiC successfully incorporate with aluminium–zinc alloy shown in SEM and XRD test. P.B. Pawar and Abhay A. Utpat (2014). Found aluminium silicon carbide composite material utilised for power transmission gears and highest hardness value obtained in 10% SiC composite material. H.M. Zakaria (2014) revealed that the Al/SiC metal matrix composite shows a higher density and lower corrosion resistance than pure aluminium. Arun L.R. et al. (2013) found that fly ash successfully incorporates the aluminium 6061 alloys giving better mechanical properties. S.B. Hassan et al. (2013) found that the addition of 12. wt % carbonised eggshell reinforced with Al-Cu-Mg alloy gave better results in physical and mechanical properties than uncarbonised eggshell particles. Pruthviraj R.D. (2011) inferred that adding a small amount of SiC material reinforced aluminium plays a significant change in mechanical properties [11,12,13,14,15]. Iftikhar Ahmed and Prakash A. Mahanwar (2010) enhanced composite with better strength and elongation with fly ash particles. Based on the literature survey, it can be concluded that a very few authors have utilised the aluminium alloy 6082 reinforced with nitrides of Si, Mg, etc., and compare the results with Ansys platform. However, here in this research work, the hybrid form of metal matrix composite has been generated with the addition of silicon nitride (Si_3_N_4_), eggshell, and magnesium with prescribed weight ratios. The investigation will be carried out among the mechanical characteristics such as hardness, tensile strength, and compressive strength with the addition of reinforcement of Si_3_N_4_, eggshell, and magnesium (2 + 5 + 1) %. This can evaluate the mechanical properties of composites combinations through the Rockwell hardness testing (HRC) and universal testing machine (UTM). Furthermore, the influence parameters of machining conditions on surface roughness (Ra), material removal rate (MRR) and machining time of the turning process in CNC machining will be analysed. This could be analysed with the process of the Taguchi method. In addition, the ANSYS platform is used to forecast the results of mechanical testing analysis based on stresses and displacements.

## 2. Materials and Methods

**Figure 1:**
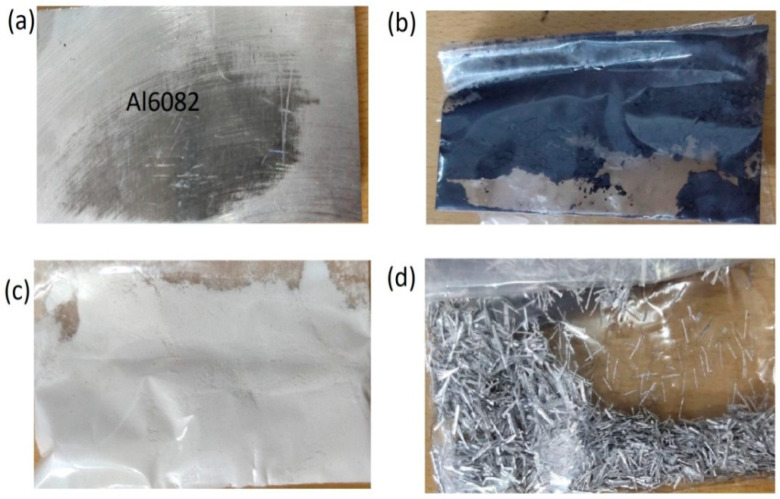
Photograph of; (**a**) Al6082, (**b**) Si_3_N_4_, (**c**) eggshells powder, (**d**) magnesium.

The material utilised was an aluminium Al6082 alloy with a minimum purity of 96.55 percent, as reported in Table 1. Silicon nitride (Si_3_N_4_) reinforcement consists of 50 nm powder particles shown in Figure 1b. Eggshells from local households were collected and then washed with demineralised water to remove any foreign objects and the thin outer membrane. The eggshells were then exposed to the sun for 48 h. Using a grain miller rotating at 250 rpm, the dried eggshells were subsequently ground into a fine powder in Figure 1c. After cooling to normal temperature, the eggshells were ground into a powder using a grain miller following one hour of heating in a 750 °C furnace. To obtain particles with a homogeneous size distribution, the resultant powder was passed through sieves of the specified mesh size.

### 2.1. Aluminium 6082

As illustrated in Figure 1, the aluminium alloy 6082 alloy has been one of the max strength alloys in the 6000 series of alloys and is one of the high strength alloys (a). In addition to having a superior surface polish, it has excellent corrosion resistance and is easily anodised. It contains a high concentration of manganese and magnesium components, as shown in Table 1, which provides a rich alloying effect. Aluminium alloy 6082 is primarily used in high-stress applications such as crane truss work, bridge construction, ore skip construction, and storage usage, including liquor barrels, milk churns, and other similar items.

### 2.2. Silicon Nitride

Silicon nitrides (Si_3_N_4_) are a scope of advanced engineering production described by high strength, toughness, hardness, and excellent chemical and thermal substances. Figure 1b shows a Silicon Nitride has the most adaptable combination of mechanical, thermal, and electrical properties of any specialised ceramic material. It outperforms most metals’ high-temperature abilities and has a predominant blend of creep and oxidation opposition. Furthermore, its low, warm conductivity and high wear obstruction make it a superb material that can withstand the most demanding conditions in the most requesting mechanical applications. Silicon nitride is an astounding decision when high-temperature and high-load capacities are required [16,17,18].

### 2.3. Eggshell

Eggshells are a substantial source of food waste, and as a result, their disposal is a serious environmental concern. There are only a few options for egg disposal. An example is shown in Figure 1c. Eggshells are biomaterials that are composed of 94–97 percent calcium carbonate. CaCO_3_ has a smaller range than synthetic calcium carbonate. CaCO_3_’s ability to serve as an excellent source of reinforcement in MMCs has led to its widespread use. Preparing eggshell powder is a time-consuming process. After rinsing the eggshells in water to remove the membrane, they were placed on a circular stainless steel tray and dried in the sun for 6 h after being cleaned. Manual crushing of the dry eggshells was accomplished with the use of hand compression, and then fine pulverisation was accomplished in a planetary ball mill for three hours before being removed from the mill [19,20,21,22].

### 2.4. Magnesium

Magnesium ranks eighth on the list of the most plentiful elements in the universe. In massive, aged stars, it is created by the consecutive inclusion of three helium nuclei to a carbon nucleus, a process known as the triple addition. Magnesium is found in the greatest abundance element in the Earth’s crust and the fourth greatest abundant element on the planet (after iron, oxygen, and silicon), accounting for 13 percent of the Earth’s total mass (see Figure 1d).

### 2.5. Preparation of Composite

**Figure 2:**
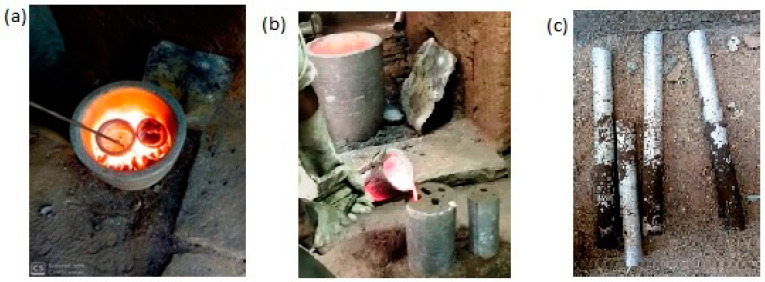
Photograph of; (**a**) crucible casting process (**b**) pouring in pattern, (**c**) cast work piece.

For manufacturing metallic components, casting is one of the manufacturing techniques to prepare. In this work, Figure 2 shows the preparation of metal matrix composites by crucible casting techniques. Table 2 shows the preparation of the aluminium mixing ratio of composite material. The aluminium alloy in the form of plates, eggshell, silicon nitride, and magnesium in powders. Aluminium plates are cleaned and melted using a crucible furnace at an appropriate temperature [23,24,25].

### 2.6. Experimentation

**Figure 3:**
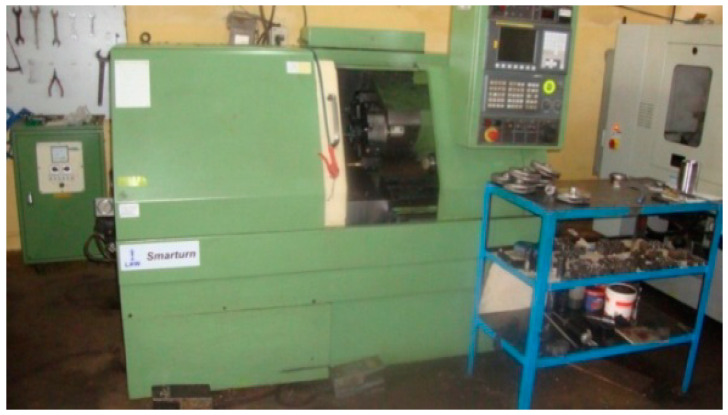
CNC Turning Machine (BATLIBOI smart run CNC lathe).

After analysing the mechanical properties further, it can be analysed through CNC turning operation with the Taguchi method’s L9 orthogonal array. Figure 3 shows that turning operation is performed using BATLIBOI smart run CNC lathe and reinforced composite material as a workpiece material of length 60 mm and 26 mm diameter. Tool material Carbide-TAEGUTEC-TT-5100-04 insert used for experimental study. Machining time, material removal rate, and surface roughness are examined at various input parameters such as feed rate, speed, and depth of cut, as illustrated in the table.

In order to decide the most appropriate parameter for the results, Taguchi’s methodology reveals experimental and qualitative perceptions. The Taguchi robust design is a powerful method for reducing the number of experiments. Based on the L9 orthogonal array turning operation carried out using CNC shown in Table 3 and Table 4, the machining time was calculated using a stopwatch. Surface roughness was examined using surface roughness tester TR-200, and MRR was calculated using the weight method.

## 3. Results and Discussion

### 3.1. Evaluation of Mechanical Properties

With the Al matrix alloy, the hardness of AMC increased by up to 13% in terms of Si_3_N_4_ and ES particles. The hardness increased from 76 HRB at 0% to 87 HRB at 5% Si_3_N_4_ and 2% eggshell. According to Soutrik Bose et al. [13], 60 BHN at 0% to 101 BHN at 12.5%. However, increasing the wt. percent of SSA above 7.5 wt. percent results in a decrease in hardness due to a constant level. The increases were caused by an increase in the hard, brittle phases of ES in the Al alloy. However, when compared to Vikas Verma et al. [14], it is clear that increasing the wt. percent of eggshell increases the hardness of the composite in brittle fracture.

Tensile strength increases by up to 13% with the addition of 5% Si_3_N_4_ and 5% ES to the Al alloy. Tensile strength increased from 89.24 N/mm^2^ at 0% to 102.01 N/mm^2^ at 5% Si_3_N_4_ and 2% eggshell. This could be due to the formation of a nearly uniform distribution of silicon nitride and eggshell particles in the aluminium alloy matrix. It was compared to Vikas Verma et al. [14], who clearly demonstrated that increasing the addition of 5% SiC and 5% Es increases the tensile strength up to 8% but is less than 5% Si_3_N_4_ and 5% ES at the same combination fraction.

The addition of 5% Si3N4 and 5% ES increased compression strength to 59 N/mm^2^. Compression strength increases from 252.47 N/mm^2^ at 0 wt. percent to 311.4 N/mm^2^ at 5 wt. percent Si_3_N_4_ and 5 wt. percent ES, with compression load increasing up to 5 KN. According to the static analysis of mechanical testing, the maximum stress cumulating minimum displacement can withstand the Al- Si_3_N_4_-Es-Mg composite with more deviations. This can be demonstrated to be more appropriate for ductile-rich applications [22].

### 3.2. Mechanical Characterisations

The mechanical testing has been conducted by using the well-calibrated Universal Testing Machine (UTM 60 M/c 6/2007.3672) for measuring compressive and tensile strength, and hardness has been measured using the Rockwell hardness tester.

#### 3.2.1. Tensile Test

**Figure 4:**
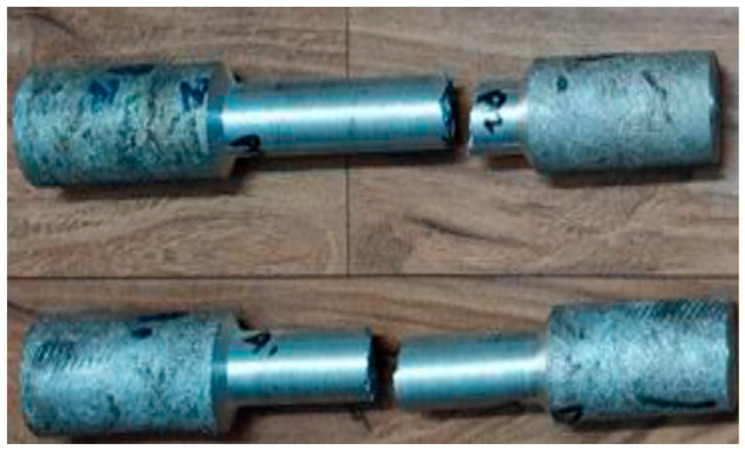
Tensile strength specimen image.

**Figure 5:**
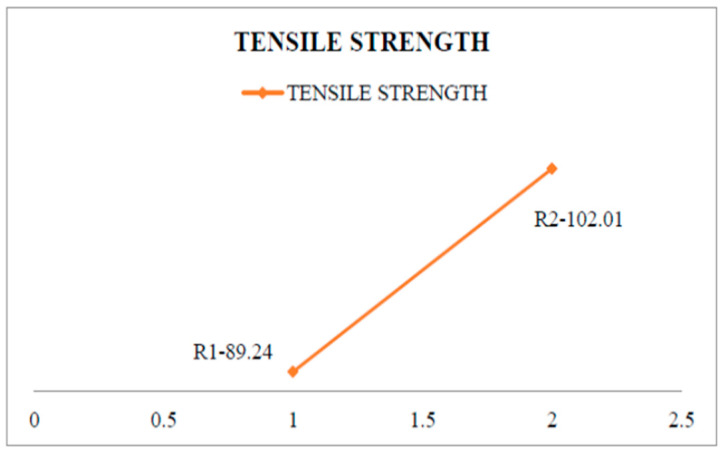
Tensile strength graph.

By way of tensile inspection, aluminium composites are prepared as per the dimension of ASTM B557M-02, a standard and the test specimen as shown in Figure 4. Table 5 shows the tensile strength test value which helps to assess tensile characteristics such as tensile strength, yield strength, elongation percentage, area reduction percentage, as well as elasticity modulus. The tensile strength can be measured with the material failure for the maximum tensile load of 17.46 KN for the obtained fracture due to tensile action over the pure aluminium. Likewise, it can be withstanding in tensile testing up to 20.57 KN tensile load. Figure 5 shows that aluminium composites material has been attained superior tensile strength compared to pure aluminium [23].

**Figure 6:**
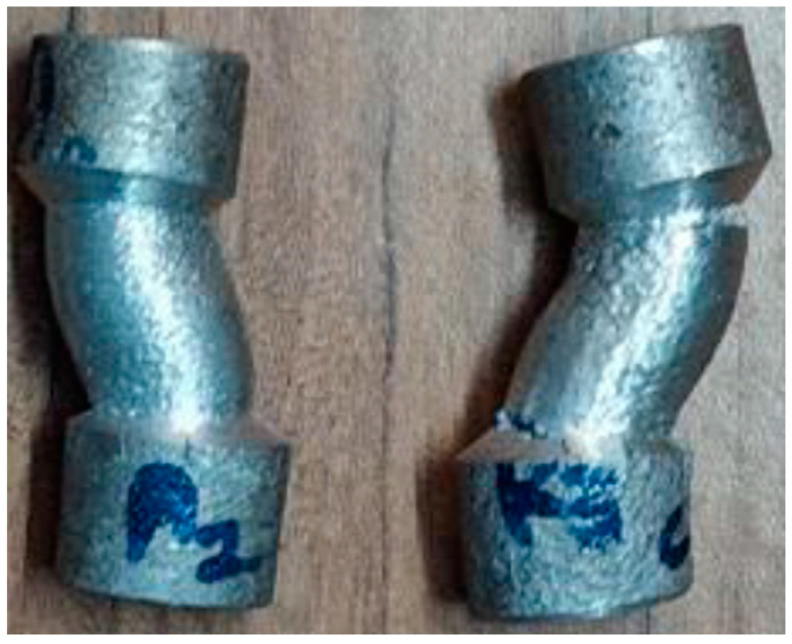
Compression strength specimen image.

**Figure 7:**
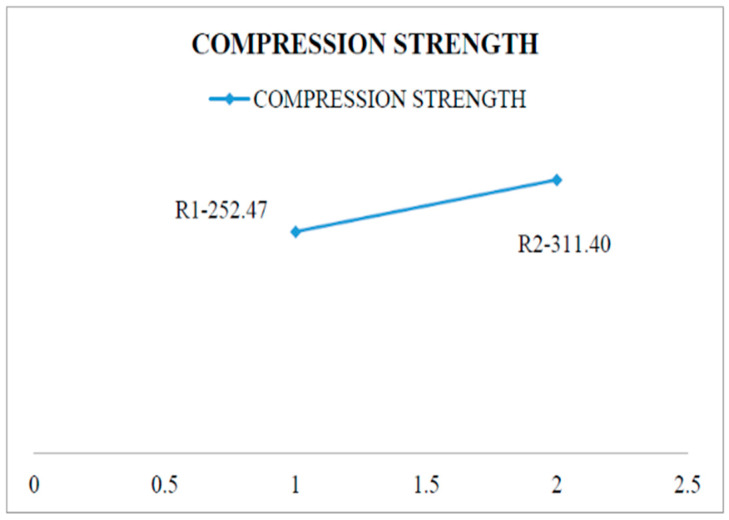
Compression strength graph.

A compression test is one in which a sample faces conflicting forces that drive from opposite sides inwards on the specimen compression. Preparation of composite material as per the dimensions of ASTM B557M-02a standards and test specimen as shown in Figure 6. The specimen has been compressed and bears up to the compressive load of 58.96 KN for the pure alloy Al6082 and the maximum load withstanding 64.29 KN has been obtained for the composite structure during the compression test as mentioned in Table 6. A compression test aims to assess the reaction of material when undergoing a compressive load. Figure 7 shows that aluminium composite material has attained higher compression strength compared to pure aluminium [24].

#### 3.2.2. Hardness Test

The hardness has been measured using 100 Kgf. load using 1/16′ ball indenter for obtaining the values of 76HRB for the pure alloy and 87HRB for the developed aluminium composite (92% Al6082 + 5% Si_3_N_4_ + 2% eggshell + 1% magnesium). The 13% hardness improvement in the aluminium composite, which was made by the casting process, is shown in Table 7 [21].

### 3.3. Statistical Analysis

Table 8 shows the final response and S/N ratio for the essential parameters such as machining time, surface roughness, and material removal rate and is exposed in a graphical format to understand the input parameters’ results.

#### 3.3.1. Taguchi Analysis: Machining Time versus SPEED, FEED, DOC

Table 9 shows the response table for the S/N ratio and means for machining time. The optimum process parameters obtained from the response values are A2 B2 C3 for the table control factors. The optimum process parameter of machining time concludes that speed and feed rate at level 2 and depth of cut at level 3 [24]. The experimental results of the composite materials were analysed with the perception of “Smaller is Better”.

**Figure 8:**
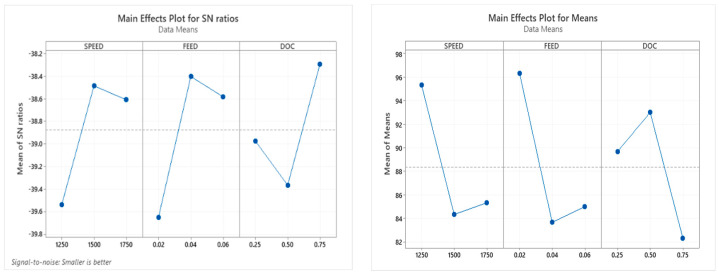
Main effect plot for SN ratio and means for machining time.

Figure 8 shows the main effect plot for the S/N ratio and means for machining time. The effect of speed increases from 1250 to 1500 rpm and decreases from 1500 to 1750; the optimum machining time is 1500 rpm. The rate of feed rises from 0.02 to 0.04 mm/rev and decreases from 0.04 to 0.06. So, the 0.04 feed rate shows the optimum depth of the cut decreases from 0.25 to 0.50 mm and suddenly increases from 0.50 to 0.75 mm, so the optimum depth of the cut is 0.75 mm [25].

#### 3.3.2. General Linear Model: Machining Time versus Spindle Speed, Feed, Depth of Cut

ANOVA identifies the process parameters that influence the characteristics of composite materials. The ANOVA results with the percentage of contribution for machining time as mentioned in Table 10. The major contribution is the feed rate at a confidence level of 40.05%, followed by the depth of cut of 29.84% and a spindle speed of 25.85%. The error has been chronicled as 4.26%, respectively, justifying that the error is the least [23].

#### 3.3.3. Taguchi Analysis: RA versus SPEED, FEED, DOC

Table 11 shows the response table for the S/N ratio and means for surface roughness. The optimum process parameters obtained from the response values are A2 B1 C2 for the table control factors. The optimum process parameter of surface roughness concludes that speed and depth of cut at level 2 and feed rate at level 1. The experimental results are the perception of “Smaller is Best”.

**Figure 9:**
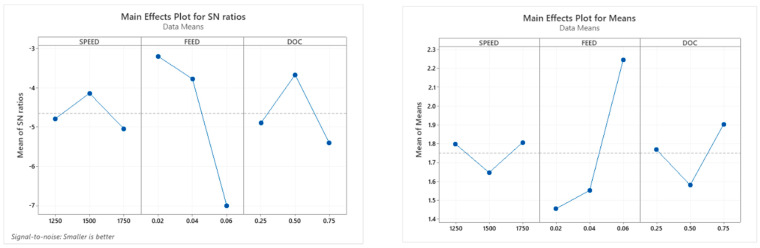
Highest influence plot for SN ratio and means for Ra.

Figure 9 illustrates that the foremost effect plot for S/N ratio and means for surface roughness, the main effect of speed increases from 1250 to 1500 rpm and decreases from 1500 to 1750; the optimum machining time is 1500 rpm. The feed rate decreases from 0.02 to 0.04 mm/rev and suddenly decreases from 0.04 to 0.06 so that the 0.02 feed rate shows the optimum value. The depth of the cut increases from 0.25 to 0.50 mm and suddenly decreases from 0.50 to 0.75 mm, so the optimum depth of the cut is 0.50 mm [21].

#### 3.3.4. General Linear Model: RA versus Spindle Speed, Feed, Depth of Cut

Table 12 shows the ANOVA outcomes for surface roughness. The major contribution is the feed rate at a confidence level of 79.73%, trailed by the depth of cut of 13.01% and a spindle speed of 3.71%. The error has been recorded as 3.56%, respectively, justifying that the error is least and highly reliable.

#### 3.3.5. Taguchi Analysis: MRR versus SPEED, FEED, DOC

Table 13 provides the final response and S/N ratio for the material removal rate. The practical consequences are the observation of “Larger is Better”. The feed rate exposed a significant contribution to achieving the maximum material removal rate followed by the depth of cut act as the second major process parameter in this optimisation.

**Figure 10:**
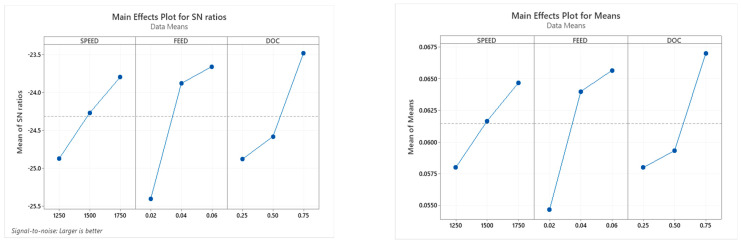
Main effect plot for SN ratio and means for MRR.

Figure 10 shows the main effect plot for the S/N ratio and means for MRR. The effect of speed increases from 1250 to 1750 rpm; the optimum machining time is 1750 rpm. The feed rate increases from 0.02 to 0.04 mm/rev and gradually increases from 0.04 to 0.06 mm/rev. The feed rate at 0.06 mm/rev shows the optimum value. The depth of the cut increases from 0.25 to 0.75 mm, and so the optimum depth of the cut is 0.75 mm [25].

#### 3.3.6. General Linear Model: MRR versus Spindle Speed, Feed, Depth of Cut

Table 14 shows the ANOVA results for material removal rate. The major contribution is the feed rate at a confidence level of 36.83%, followed by the depth of the cut at 26.43% and a spindle speed of 11.87%. The R square for the model is 74.32%, respectively.

#### 3.3.7. Analysis of Stresses and Displacements for UTM Outcomes

**Figure 11:**
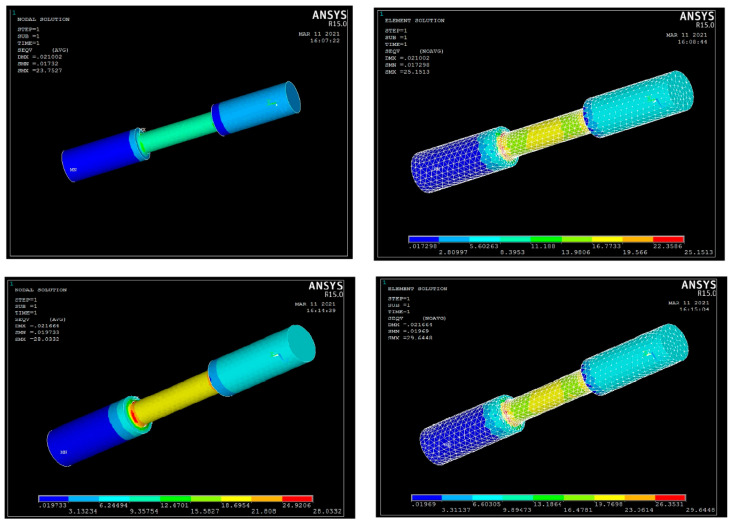
Analysis of nodal and elemental solution plot for tensile test on pure Al and composite in ANSYS.

**Figure 12:**
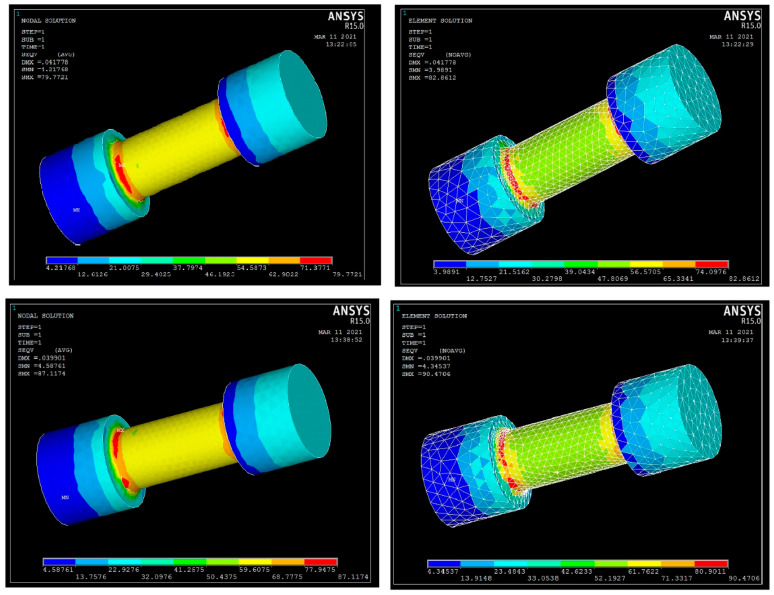
Analysis of nodal and elemental solution plot for compression test on pure Al and composite in ANSYS.

**Figure 13:**
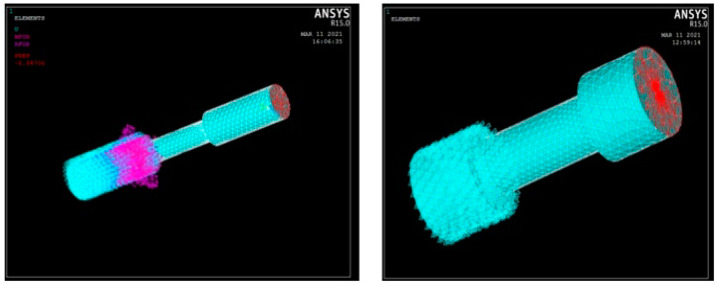
Convergence mesh generation on IGES format specimen model.

For predicting the performance of the composite specimen as well alloy components, the UTM specimen model has been generated using the Pro CREO modelling platform as per the required geometrics. The Initial Graphics Exchange Specification (IGES) format specimen model has been imported and the material properties have been allotted in the ANSYS ADSL platform. The nodal and elemental solution plot for tensile test on pure aluminium and composite aluminium using ANSYS is shown in Figure 11. In this ANSYS simulation, the solid model of the required specimen has been imported in IGES format for measuring the stresses and deformation during the compression and tension. The element type used here is Brick 8 node and the tetrahedral mesh type is also used for the mesh generation. The number of nodes was recorded as 2671 and the elements are 10,855 through the simulation for the element size factor of 4. The complete mesh type is occupied with the surface polygons of 3020. Likewise, nodal and elemental solution analysis for the compression test is shown in Figure 12. The convergence mesh was generated over the specimen before the application of displacement and load conditions, as shown in Figure 13. The static conditional simulation was assigned, followed by the displacement, which was set to be zero at the fixed side. Table 15 shows that Young’s modulus, density, and Poisson’s ratio for aluminium, composite material, and load have been applied in the form of pressure on the surface of another flat end which was connected with the upper or lower jaw of the UTM machine when experimentation [24]. The dynamic conditions of any machine or machining components can withstand stress whether it may be in static or dynamic conditions. Based on the support systems and load acting positions, the tensile and compressive action of forces acting on any element can be easily predicted through the simulation platform. Based on the fixed end of the boundary conditions and load acting end can be easily defined in the simulation software. The material properties can be defined through the values of Young’s modulus and Poisson’s ratio with the consideration of volume density. So, the simulation procedures helps to predict the stresses or strain acting over the specimen which has to be proved its fertility of ductile characteristics during the tension or in compression.

Table 16 shows the differentiation of simulation regarding compression and tensile test outcomes of pure aluminium alloy Al6082 and the aluminium composite (92% Al6082 + 5% Si_3_N_4_ + 2% eggshell + 1% magnesium). The nodal stress and elemental stress have been carried out with the help of the ANSYS platform. The stress can be predicted using the comparative method of finding and comparing the simulative outcomes and the experimental validation data of tensile and compression tests.

**Figure 14:**
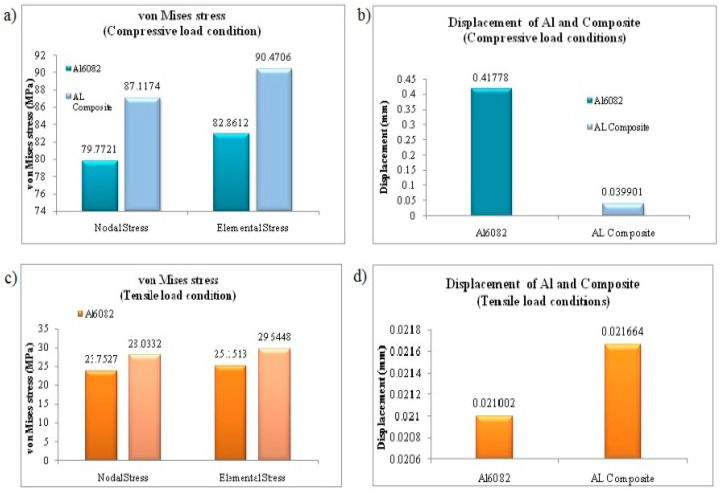
(**a**) Comparison of von Mises stress under compressive test, (**b**) displacement values of al and composite compressive load conditions, (**c**) nodal and elemental solution of von Mises stress under tensile load conditions, (**d**) comparison of nodal displacements under tensile load conditions.

As depicted in Table 17, the nodal displacements and failure stress (von Mises) can be compared for different composite materials based on the tensile and compressive load conditions. The displacement values are also obtained in Table 17. Here, the stress values were attained for the compressive test and the tensile action for the specimen dimensions of 60 × 25 mm for compressive and 200 × 25 mm for tensile at various load conditions [18]. By the consideration of the compressive test, the von Mises stress was attained over the nodal points at 87.1174 MPa and for the elemental stress at 90.4706 MPa for the aluminium composite (92% Al6082 + 5% Si_3_N_4_ + 2% eggshell + 1% magnesium) as shown in Figure 14a. By the consideration of the tensile test, the von Mises stress was attained over the nodal points at 28.0332 MPa and for the elemental stress at 29.6448 MPa for the aluminium composite as shown in Figure 14b.

Comparatively, lesser stress values were obtained for the pure Al6082 alloy, as mentioned in Figure 14b. A comparison has been made among the displacement values of the composite and the pure alloy, which shows lesser displacement using the aluminium composite of 0.039901 mm for the compressive test as mentioned in Table 17. The same range of displacements has occurred for the tensile test as mentioned in Figure 14d.

### 3.4. Interpretation of Results

In this research work, the mechanical properties have been evaluated on the basis of hardness findings. The better hardness was achieved for the composites because of the well-bonded grain structure with the incorporation of Si_3_N_4_ and minimal contributions of eggshell and magnesium. The performance of mechanical testing has been predicted with the outcomes of stresses and displacements using the ANSYS platform. Due to the higher hardness and high stress, the values of compressive and tensile testing confirmed that the prepared composite was achieved better. This can be measured and confirmed as better results using graphical results. The stir casting method of the composite preparation was accomplished, and by performing mechanical testing such as hardness, tensile, and compression, there are no visible defects within the specimen upon visual inspection. This specimen has been involved in turning operations [19]. Optimisation results much benefited the identification of the influencing factor of feed rate as the primary factor on MRR, SR, and machining time has been confirmed. In addition, the second factor of the depth of the cut has influenced surface roughness a little bit.

## 4. Conclusions

Thus, it has been found that silicon nitrate and eggshell (Si_3_N_4_ and Es) have been successfully incorporated in Al6082 matrix alloy through the crucible casting technique. Composite materials, especially aluminium 6082 and silicon nitrate, eggshell and magnesium composites have achieved excellent mechanical characteristics compared with the conventional materials. It can be used for various industrial applications, which are light in weight and high hardness. From the investigation, the mechanical properties of the Al6082 metal matrix have been analysed with an outcome of tensile, hardness, and compressive strength. It has found that Ratio 2 (Al6082 + Si_3_N_4_ − 2% + Es − 5% + Mg − 1%) has been attained superior tensile, hardness and compressive strength compared with a pure alloy of Al6082.

In this experimentation, the ANOVA method of Taguchi optimisation was utilised to obtain the optimal turning parameters in the metal matrix composite. The present work investigated roughness, machining time, and metal removal rate with the influencing parameters of CNC turning using coated carbide inserts. Based on the investigations, the following conclusions have been made.

- Better surface quality of about 1.217 μm roughness was obtained during the turning operation. It is noted that the minimum surface roughness was obtained for the depth of cut of 0.50 mm, spindle speed of 1500 RPM, and feed rate of 0.02 mm/rev, respectively.
- Less machining time of 76 s was achieved at the spindle speed of 1500 RPM, depth of cut of 0.75 mm, and feed rate of 0.04 mm/rev, respectively.
- A higher material removal rate of about 0.069 gm/cc was obtained for the depth of cut of 0.75 mm, spindle speed of 1750 RPM, and feed rate of 0.02 mm/rev, respectively.
- Feed rate has considered the most dominant factor for all output responses. The highlighted values are identified with the percentage of contribution of feed rate on surface roughness as 80%, the machining timing as 40% and the material removal rate contributed as 37%.

Based on static analysis of mechanical testing, the maximum stress cum minimum displacement can withstand the Al-Si_3_N_4_-Es-Mg composite with more deviations. This can be proved that this could be more suitable for ductile-rich applications.

## Figures and Tables

**Table 1:** Constraints of chemical composition.

Weight %	Al	Si	Fe	Cu	Mn	Cr	Mg	Zn	Ti	Other Each	Others Total
6082	Bal	0.7–1.3	0.50 max	0.10 max	0.40–1.00	0.25 max	0.06–1.20	0.20 max	0.10 max	0.05 max	0.15 max

**Table 2:** Mixing ratio of composite.

Ratio	AL 6082 Wt (%)	Si_3_N_4_ Wt (%)	ES Wt (%)	Mg Wt (%)
1	100%	0	0	0
2	92%	5%	2%	1%

**Table 3:** Identifying process parameters and their corresponding levels.

Levels	Machining Parameters		
	Spindle Speed (N) (RPM)	Feed (mm/Rev)	DOC (mm)
1	1250	0.02	0.25
2	1500	0.04	0.50
3	1750	0.06	0.75

**Table 4:** Experimental design (L9 orthogonal array).

S. No	Spindle Speed (N) (RPM)	Feed (mm/Rev)	DOC (mm)
1	1250	0.02	0.25
2	1250	0.04	0.50
3	1250	0.06	0.75
4	1500	0.02	0.50
5	1500	0.04	0.75
6	1500	0.06	0.25
7	1750	0.02	0.75
8	1750	0.04	0.25
9	1750	0.06	0.50

**Table 5:** Tensile strength test value.

Sample	YL (kN)	YS (N/mm^2^)	TL (kN)	TS (N/mm^2^)	IGL (mm)	FGL (mm)	% E	FD	% RA
R1—Al6082-100%	14.57	74.47	17.46	89.24	50.00	51.24	2.48	15.06	8.92
R2—92% Al6082 + 5% Si_3_N_4_ + 2% Eggshell + 1% Magnesium	15.92	78.95	20.57	102.01	50.00	51.34	2.68	15.41	7.47

**Table 6:** Compression strength test value.

Sample	Compression Load, kN	Compressive Strength, N/mm^2^
R1—Al6082-100%	58.96	252.47
R2—92% Al6082 + 5% Si_3_N_4_ + 2% Eggshell + 1% Magnesium	64.29	311.40

**Table 7:** Hardness value.

Sample	HRB
R1—Al6082-100%	76
R2—92% Al6082 + 5% Si_3_N_4_ + 2% Eggshell + 1% Magnesium	87

**Table 8:** Orthogonal array (L9) with S/N ratio for machining time, surface roughness, MRR.

Sl. No.	Speed (A)	Feed (B)	DOC (C)	Machining Time	Ra	MRR	S/N Ratio for Machining Time	S/N Ratio for Ra	S/N Ratio for MRR
	(RPM)	(mm/Rev)	mm	SEC	Micron	gm/cc			
1	1250	0.02	0.25	107	1.588	0.044	−40.5877	−4.01701	−27.1309
2	1250	0.04	0.5	96	1.328	0.064	−39.6454	−2.46396	−23.8764
3	1250	0.06	0.75	83	2.479	0.066	−38.3816	−7.88553	−23.6091
4	1500	0.02	0.5	94	1.217	0.051	−39.4626	−1.70581	−25.8486
5	1500	0.04	0.75	76	1.669	0.066	−37.6163	−4.44913	−23.6091
6	1500	0.06	0.25	83	2.056	0.068	−38.3816	−6.26046	−23.3498
7	1750	0.02	0.75	88	1.563	0.069	−38.8897	−3.87918	−23.223
8	1750	0.04	0.25	79	1.661	0.062	−37.9525	−4.40739	−24.1522
9	1750	0.06	0.5	89	2.197	0.063	−38.9878	−6.8366	−24.0132

**Table 9:** Response table for machining time.

Response Table for Signal to Noise Ratios				Response Table for Means			
Smaller Is Better							
Level	SPEED	FEED	DOC	Level	SPEED	FEED	DOC
1	−39.54	−39.65	−38.97	1	95.33	96.33	89.67
2	−38.49	−38.4	−39.37	2	84.33	83.67	93
3	−38.61	−38.58	−38.3	3	85.33	85	82.33
Delta	1.05	1.24	1.07	Delta	11	12.67	10.67
Rank	3	1	2	Rank	2	1	3

**Table 10:** Analysis of variance for transformed response for machining time.

Source	DF	Seq SS	Contribution	Adj SS	Adj MS	F Value	*p* Value
Spindle Speed	2	173.25	25.85%	173.25	86.63	6.07	0.141
Feed	2	268.45	40.05%	268.45	134.23	9.41	0.096
Depth of Cut	2	199.99	29.84%	199.99	99.99	7.01	0.125
Error	2	28.54	4.26%	28.54	14.27		
Total	8	670.23	100.00%				

**Table 11:** Response table for Ra.

Response Table for Signal to Noise Ratios					Response Table for Means		
Smaller Is Better							
Level	SPEED	FEED	DOC	Level	SPEED	FEED	DOC
1	−4.789	−3.201	−4.895	1	1.798	1.456	1.768
2	−4.138	−3.773	−3.669	2	1.647	1.553	1.581
3	−5.041	−6.994	−5.405	3	1.807	2.244	1.904
Delta	0.903	3.794	1.736	Delta	0.16	0.788	0.323
Rank	3	1	2	Rank	3	1	2

**Table 12:** Analysis of variance for transformed response for Ra.

Source	Seq SS	Contribution	Adj SS	Adj MS	F Value	*p* Value
Spindle Speed	0.007014	3.71%	0.007014	0.003507	1.04	0.49
Feed	0.150814	79.73%	0.150814	0.075407	22.39	0.043
Depth of Cut	0.024601	13.01%	0.024601	0.012301	3.65	0.215
Error	0.006737	3.56%	0.006737	0.003368		
Total	0.189166	100.00%				

**Table 13:** Response table for MRR.

Response Table for Signal to Noise Ratios				Response Table for Means			
Higher Is Better							
Level	SPEED	FEED	DOC	Level	SPEED	FEED	DOC
1	−24.87	−25.40	−24.88	1	0.05800	0.05467	0.05800
2	−24.27	−23.88	−24.58	2	0.06167	0.06400	0.05933
3	−23.80	−23.66	−23.48	3	0.06467	0.06567	0.06700
Delta	1.08	1.74	1.4	Delta	0.00667	0.011	0.009
Rank	3	1	2	Rank	3	1	2

**Table 14:** Analysis of variance for transformed response for MRR.

Source	DF	Seq SS	Contribution	Adj SS	Adj MS	F Value	*p* Value
Spindle Speed	2	0.000067	11.87%	0.000067	0.000033	0.46	0.684
Feed	2	0.000211	36.83%	0.000211	0.000105	1.46	0.407
Depth of Cut	2	0.000142	26.43%	0.000142	0.000071	0.98	0.506
Error	2	0.000145	24.87%	0.000145	0.000072		
Total	8	0.000564	100.00%				

**Table 15:** Mechanical testing outcomes of tensile and compressive pressures.

Material/Mechanical Properties	Young’s Modulus (GPa)	Density (gm/cc)	Poisson Ratio	Pressure (N/mm^2^)	
Al6082-100%	71	2.71	0.33	Tensile	23.1216
				Compressive	6.847059
92% Al6082 + 5% Si_3_N_4_ + 2% Eggshell + 1% Magnesium	81.131	2.7179	0.326	Tensile	25.2118
				Compressive	8.0666

**Table 16:** Simulation outcomes of nodal and elemental stress on Al6082 and Al + ES + Mg composite.

Samples	Compression Test		Tensile Test	
	Nodal Stress (MPa)	Elemental Stress (MPa)	Nodal Stress (MPa)	Elemental Stress (MPa)
Al6082-100%	79.7721	82.8612	23.7527	25.1513
92% Al6082 + 5% Si_3_N_4_ + 2% Eggshell + 1% Magnesium	87.1174	90.4706	28.0332	29.6448
Difference	8.43	8.41	15.27	15.16

**Table 17:** Nodal displacements of Al6082 and Al composite during compression and tensile.

Samples	Compression Test Displacement (mm)	Tensile Test Displacement (mm)
Al6082-100%	0.41778	0.021002
92% Al6082 + 5% Si_3_N_4_ + 2% eggshell + 1% magnesium	0.039901	0.021664

## Data Availability

The data used to support the findings of this study are included within the article.
